# The Use of Cardioprotective Devices and Strategies in Patients Undergoing Percutaneous Procedures and Cardiac Surgery

**DOI:** 10.3390/healthcare11081094

**Published:** 2023-04-11

**Authors:** Toufik Abdul-Rahman, Ileana Lizano-Jubert, Neil Garg, Emilio Tejerina-Marion, Syed Muhammad Awais Bukhari, Ana Luisa Ek, Andrew Awuah Wireko, Adriana C. Mares, Vladyslav Sikora, Rahul Gupta

**Affiliations:** 1 Medical Institute, Sumy State University, 40007 Sumy, Ukraine; drakelin24@gmail.com (T.A.-R.); andyvans36@yahoo.com (A.A.W.); v.sikora@med.sumdu.edu.ua (V.S.); 2 Department of Cardiology, Otto Von Guericke University of Magdeburg, 39120 Magdeburg, Germany; 3 Centro de Investigación en Ciencias de la Salud (CICSA), FCS, Universidad Anáhuac Campus Norte, Huixquilucan 52786, Mexico; ileana1500@gmail.com (I.L.-J.); emiteje2000@gmail.com (E.T.-M.); analuisa.ek2002@gmail.com (A.L.E.); 4 Rowan-Virtua School of Osteopathic Medicine, One Medical Center Drive Stratford, Stratford, NJ 08084, USA; gargne36@rowan.edu; 5 Department of Medicine, Nishtar Medical University, Multan 60000, Pakistan; drawaisbukhari301@gmail.com; 6 Division of Cardiovascular Medicine, Texas Tech University Health Sciences Center El Paso, El Paso, TX 79905, USA; acvmares@yahoo.com; 7 Department of Clinical and Experimental Medicine, University of Foggia, Via Napoli, 20, 71122 Foggia, Italy; 8 Department of Cardiology, Lehigh Valley Health Network, Allentown, PA 18103, USA

**Keywords:** cardiac surgery, ventricular assist devices, ischemia–reperfusion injury

## Abstract

In the United States, about one million people are seen to visit the operating theater for cardiac surgery annually. However, nearly half of these visits result in complications such as renal, neurological, and cardiac injury of varying degrees. Historically, many mechanisms and approaches have been explored in attempts to reduce injuries associated with cardiac surgery and percutaneous procedures. Devices such as cardioplegia, mechanical circulatory support, and other methods have shown promising results in managing and preventing life-threatening cardiac-surgery-related outcomes such as heart failure and cardiogenic shock. Comparably, cardioprotective devices such as TandemHeart, Impella family devices, and venoarterial extracorporeal membrane oxygenation (VA-ECMO) have also been proven to show significant cardioprotection through mechanical support. However, their use as interventional agents in the prevention of hemodynamic changes due to cardiac surgery or percutaneous interventions has been correlated with adverse effects. This can lead to a rebound increased risk of mortality in high-risk patients who undergo cardiac surgery. Further research is necessary to delineate and stratify patients into appropriate cardioprotective device groups. Furthermore, the use of one device over another in terms of efficacy remains controversial and further research is necessary to assess device potential in different settings. Clinical research is also needed regarding novel strategies and targets, such as transcutaneous vagus stimulation and supersaturated oxygen therapy, aimed at reducing mortality among high-risk cardiac surgery patients. This review explores the recent advances regarding the use of cardioprotective devices in patients undergoing percutaneous procedures and cardiac surgery.

## 1. Introduction

Cardiac surgery is a lifesaving, innovative, and ever-growing field that accounts for almost one million operating room visits in the United States annually. However, 49.5% of these visits result in complications [1]. Cardiac surgery is distinct from all other sectors of percutaneous procedures due to its unique complications, management strategies, and associated injuries [1,2]. During cardiac surgery procedures, ischemia is artificially induced through aortic cross-clamping and cardioplegia via hypothermic electrolyte and sugar manipulation to minimize myocardial metabolic work, allowing clinicians to more effectively operate on the heart in its more vulnerable state [3]. At the end of the procedure, coronary flow is restored, leaving the myocardium vulnerable to the devastating effects of reperfusion injury, which is the damage caused by the re-establishment of blood supply to a tissue or organ after a period of ischemia. In addition to ischemia, patients are exposed to anesthesia ventilation, medical instruments, and other noxious stimuli [4]. These exposures can lead to severe hemodynamic events as well as inappropriate stimulation of the innate and adaptive immune systems, leading to renal injury, neurological injury, peripheral nerve injury, pulmonary complications, vasospasm, dysregulation of endothelial cell-platelet interactions, shock, and inflammation [1,2,5]. High-risk populations such as the elderly, those with a history of heart surgery or vascular/respiratory disease, and those with elevated renal markers are disproportionately affected by the adverse outcomes and injuries related to cardiac surgery [2]. These patients are particularly at risk of undergoing perioperative myocardial infarction after an acute ischemia–reperfusion injury [5]. Acute renal failure (ARF) is a clinically relevant outcome of cardiac surgery, affecting up to 30% of patients undergoing the procedure [5]. The manifestation of post-cardiac surgery, ARF is associated with considerable mortality and lifelong dialysis, especially in those patients with pre-existing risk factors [5]. Central nervous system injury is another pertinent long-term sequela of cardiac surgery injury as it is correlated with high rates of mortality and severe reduction in quality of life [6]. Cognitive deterioration post-cardiac surgery affects close to 80% of patients [6].

To prevent these adverse outcomes, percutaneous mechanical circulatory support (MCS) devices are utilized periprocedurally, intraprocedurally, and post-procedurally [7]. There exist many devices to curb the adverse effects and prevalence of cardiac procedure-related injuries. These devices act at different levels; either providing or improving hemodynamic support such as TandemHeart, Impella family devices, veno-arterial extracorporeal membrane oxygenation (VA-ECMO), intra-aortic balloon pump (IABP), etc; or by inducing cardioplegia, thus preserving myocardial properties such as topic cardioprotective cooling devices [8,9,10,11]. Although these devices and approaches have shown some promising results and are currently widely used for treating other conditions such as the use of VA-ECMO in respiratory failure, their uses in treating or preventing hemodynamic alterations in regard to cardiac surgery or percutaneous interventions still bear some important adverse effects that do not permit diminish of mortality in high-risk patients undergoing cardiac surgery or percutaneous interventions [12,13,14,15,16]. Therefore, while the use of one device over another in terms of efficacy remains controversial, further research must be conducted to assess their potential in different settings, whether that involves a single device or a combination of several [17,18,19,20,21]. Moreover, research into new strategies and targets such as transcutaneous vagus stimulation and supersaturated oxygen therapy, among others, that are being developed in order to reduce the mortality rate among high-risk patients undergoing cardiac surgery is needed [22,23]. The purpose of this review is to explore the recent discoveries regarding cardioprotective devices, their advantages and limitations in the setting of cardiac surgery, and interventional procedures for preventing or treating cardiovascular diseases in high-risk populations. Furthermore, we discuss new targets and approaches that have shown promising results in reducing mortality associated with cardiac surgery in high-risk populations.

## 2. Cardiac Surgery/Percutaneous Procedures-Related Injuries and How They Affect Ventricular Performance

**Figure 1:**
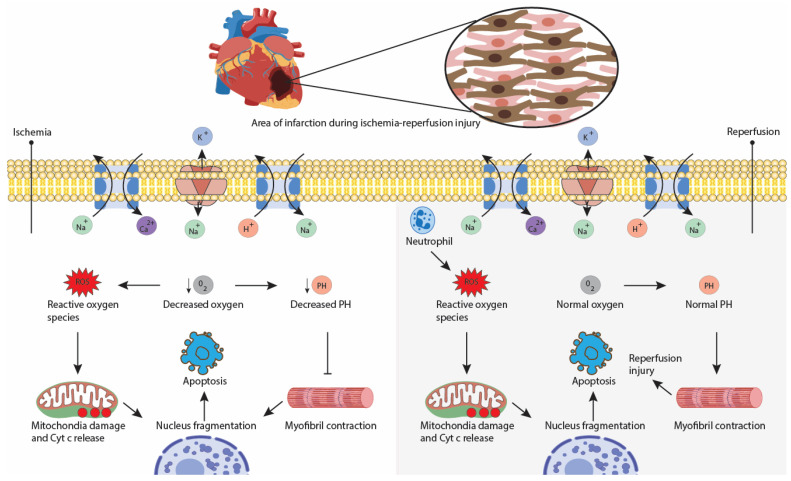
Mechanism of ischemia–reperfusion injury. Caption: During cardiac surgery, cross-clamping of the aorta induces ischemia, decreasing oxygen supply to cardiomyocytes. This increases reactive oxygen species production, decreases PH, and causes injury to cardiomyocytes. During reperfusion after surgery, diffuse inflammatory response, driven by increased reactive oxygen species production and immune cells in ischemic regions, causes further injuries and death of cardiomyocytes.

The most common cardiac-surgery-related injuries are supraventricular tachycardia (SVT), atrial fibrillation, bradycardia, atrioventricular block, cardiac arrest, reperfusion injury, and sudden cardiac death [3]. All of these pathologic states lead to altered ventricular performance and may additionally result in cardiogenic shock (CS) or congestive heart failure (CHF). They could also lead to decreased end-organ perfusion which could result in further complications and ultimately patient death [24,25]. Decreased end-organ perfusion is considered to be one of the major injury pathways related to cardiac-surgery-associated acute kidney injury (CSA-AKI), which is considered the most common clinically important complication following open heart surgery and is associated with high morbidity and mortality [25]. Furthermore, CS and CHF are considered to be the major causes of death in patients undergoing percutaneous coronary intervention (PCI) after ST-elevation myocardial infarction [26]. It is, therefore, imperative to preserve adequate cardiac output and end-organ perfusion by appropriately addressing CS or CFH to reduce patient morbidity and mortality preoperatively, intraoperatively, and postoperatively [25]. In regards to the use of PCI after acute myocardial infarction (AMI), reperfusion of the myocardium may also lead to reperfusion injury [3]. In this setting, reperfusion of the myocardium leads to a diffuse inflammatory response driven by increased oxidative stress, accumulation of cytokines and chemical mediators, complement activation, endothelial nitric oxide release, and induction of NO synthase (Figure 1) [3,24]. Systemic microvascular injury often follows in the affected ischemic–reperfused (I/R) tissues and other organs [24]. Ventricular malfunction, organ failure, post-surgery pulmonary edema, acute respiratory failure, and sudden patient death are just some of the feared outcomes of reperfusion injury [24,27].

Arrhythmias are also common forms of cardiac-surgery-related injuries, often associated with morbidity post-cardiac surgery [28]. Although some arrhythmias can be subclinical, severe ones can lead to ventricular dysfunction, hemodynamic injury, and embolism production [28]. Ventricular performance is also mostly affected postoperatively, especially in cardio-vulnerable patients [27]. Therefore, rapid unloading of the left ventricle after surgery can lead to alteration in its size and shape, and its eventual failure [27]. In patients who are subjected to substandard periprocedural cardiac protection, prolonged cardiopulmonary bypass time, or prolonged ischemia, ventricular failure is often observed, which, in turn, results in further complications such as CS [27]. In this regard, the principle of ventricular unloading (further explained below) has been demonstrated to significantly improve cardiac function, and suggested to prevent heart failure. This may be a potential method for decreasing morbidity and mortality associated with cardiac surgery by significantly improving cardiac function [29].

## 3. Principle of Ventricular Unloading

Ventricular unloading refers to the use of any therapy, maneuver, or intervention that decreases the power expenditure of the ventricle in order to minimize myocardial oxygen consumption (MVO2), and limits the hemodynamic forces that conduct to ventricular remodeling after any injury to the heart [29].

This is based on the concept that MVO2 is directly related to power expenditure and the total amount of work performed by the heart. In other words, the oxygen requirements of the heart depend mostly on total mechanical work and energy necessary for meeting the O2 demand by the body [29,30]. The harder the heart works to meet this demand, the higher the myocardial oxygen demand and consumption. In a healthy heart, an increase in O2 requirements by the body is adequately met by the activation of compensatory mechanisms, which allow the preservation of an adequate cardiac output and mean arterial pressure (MAP), resulting in a favorable oxygen supply [30]. Conversely, when the heart is injured (i.e., AMI), the functional capacity of the heart to preserve adequate cardiac output (CO) is compromised, as the viable myocardium becomes smaller [29,30]. The small viable myocardium has to work harder to maintain a favorable end-organ oxygen supply, resulting in higher stress on the heart. If not addressed, the higher myocardial stress inevitably leads to further myocardial damage and fewer viable myocardium. This results in a feedback loop where the burden of maintaining sufficient CO is placed on a lower and lower viable myocardium [30]. Thus, compensatory mechanisms such as heart rate and heart contractility are strongly activated, resulting in a higher MVO2 that ultimately will result in heart tissue remodeling [30]. However, compensatory mechanisms including heart tissue remodeling are limited, and depending on the extent of the AMI injury, this will ultimately lead to heart failure or even cardiogenic shock. Studies have shown that ventricular unloading before, during, or after an AMI can significantly improve cardiac function post-infarction by reducing infarct size [30,31,32].

## 4. Benefits of Left Ventricular Unloading

The cardioprotective benefit of left ventricular (LV) unloading has particularly been documented in percutaneous coronary intervention for acute treatment of AMI. Studies strongly suggest that unloading the left ventricle before reperfusion (conversely to primary reperfusion) after an AMI can significantly limit infarct size [33]. More specifically, primary LV unloading 30 min before reperfusion has been proposed to significantly decrease infarct size in contrast to reperfusion alone and LV unloading 15 min before reperfusion. In biological terms and consistent with that result, LV unloading before reperfusion has been shown to downregulate the expression of genes involved in mitochondrial function and cellular respiration, thus lowering myocardial damage [32]. Moreover, stromal cell-derived factor-1α (SDF-1) and its receptor CXCR4 appear to be more elevated when LV unloading 30 min before reperfusion, compared with reperfusion alone or with LV unloading 15 min before reperfusion [32,34]. SDF-1 is a cardioprotective chemokine expressed in myocardial tissues after AMI.

Finally, LV unloading lessens proapoptotic signaling by lowering proapoptotic proteins such as BAX and active caspase-3, and increasing anti-apoptotic proteins like BCL-2 and BCL-XL [32]. By minimizing myocardial scar formation after AMI and preventing ventricular remodeling through LV unloading, heart failure can be managed or even prevented [29]. While the principle of unloading has been principally used in the treatment and prevention of acute AMI and its complications, it has also been shown to be beneficial in the management of other cardiomyopathies such as peripartum cardiomyopathy, microvascular obstruction, and reperfusion-induced arrhythmias among others [29,35].

## 5. Cardioprotective Devices That Unload the Heart:

Although the principle of LV unloading had been proposed to be beneficial for MVO2 lowering 40 years ago, it was not clinically possible to implement until the early-2000s when percutaneous ventricular assist devices for LV unloading started to develop [30]. Using a swine model of AMI and reducing LV workload with a TandemHeart device, Kapur et al. demonstrated for the first time that myocardial infarct size could be decreased by over 40% compared to reperfusion only [36]. Although there are currently many mechanical support devices (ECMO, IABP, surgical BiAVD), only two of them known as percutaneous ventricular assist devices (pVADs) are currently based on the LV unloading principle. These are TandemHeart (Livanova Inc., London, UK) and Impella (Abiomed Inc., Danvers, MA, USA) family devices. The mechanism by which pVADs work involves placing a catheter into the left ventricle LV, which draws blood and pumps it directly into circulation. This allows the reduction of workload on the heart without reducing the CO, thus preserving end-organ perfusion [29]. The use of pVADs in the setting of high-risk surgery holds various advantages, including preservation of end-organ perfusion, increasing time for decision-making regarding the best steps in management, and diminishing the burden and wear of the heart [37]. Although pVADS were originally used primarily for treating cardiogenic shock or heart failure, they are now used in surgical procedures, including ventricular tachycardia ablation and percutaneous procedures [38]. The main advantages and disadvantages of pVADS and other mechanical devices in PCI and cardiac surgery are comparatively described in Table 1.

### 5.1. Ventricular Assist Devices

#### 5.1.1. Devices That Unload the Heart

##### Tandemheart

**Figure 2:**
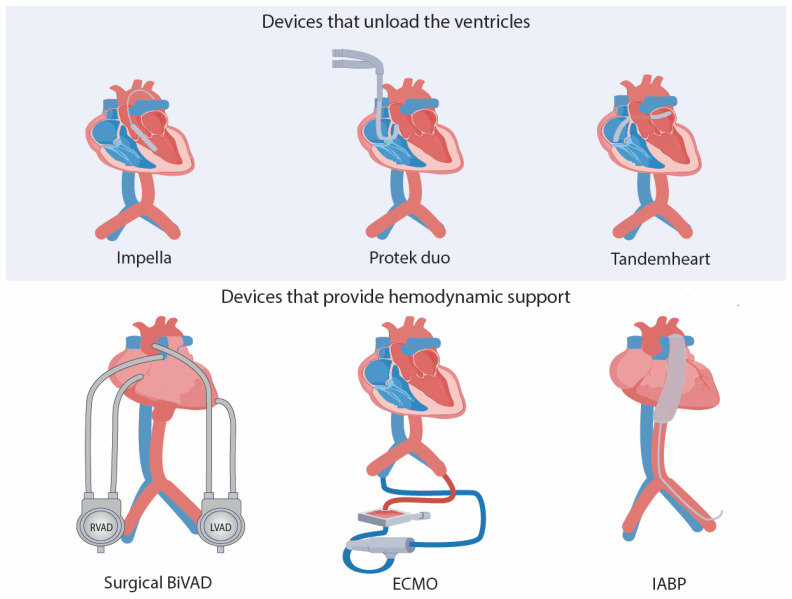
Illustration of Ventricular Assist Devices. Caption: This figure depicts the various types of ventricular assist devices used in cardiac surgery. Based on their function they are categorized into devices that unload the ventricles, and devices that provide hemodynamic support. Devices that unload the ventricles include Impella, Protekduo, and Tandemheart. Devices that provide hemodynamic support include IABP, Surgical BiVAD, and ECMO.

The TandemHeart is an external temporary device based on the insertion of a cannula (21-Fr cannula) through the interatrial septum to the left atrium. It is composed of three subsystems (a transseptal cannula, the centrifugal blood pump, and an arterial cannula) [8,39]. Oxygenated blood is pumped out from the left atrium to a centrifugal pump which continuously returns the blood to the femoral artery through a 12 Fr cannula or to the iliac artery through a 5–17 Fr cannula (Figure 2). This device is capable of generating a continuous flow of up to 4 L/min output [8,39]. Moreover, the addition of an oxygenator makes it possible for respiratory support [64]. In 2005, Thiele et al. found a statistically significant improvement in CO in patients undergoing cardiogenic shock after AMI; however, there was an increase in coagulopathy and limb ischemia with no significant difference in mortality (Table 1) [13]. Although this device is capable of pumping a good amount of blood per minute compared to other pVADs, many studies have demonstrated an increase in CO, MAP, and cardiac index. The need for puncturing the septum is an important limitation as the implantation and post-implantation challenges and risks are higher compared to other pVADS. Potential risks such as cardiac tamponade, thromboembolism, or bleeding are associated with post-transseptal puncture, and thus it delivers no improvement in mortality [13,43,65,66]. Consistent with that, a recent systematic review showed no evidence from TandemHeart of it improving survival for patients undergoing acute cardiogenic shock [67]. With regards to the use of TandemHeart in high-risk PCI, a study from the Mayo Clinic demonstrated the safety and feasibility of this device on 54 patients undergoing high-risk PCI [68]. A 97% success rate was achieved with a hemodynamic improvement during the procedure, and an 87% survival rate 6 months after the procedure. Nevertheless, 13% of patients suffered from major vascular complications [69]. In another study involving 13 patients, with 92% suffering from severe congestive heart failure symptoms, TandemHeart was placed before high-risk PCI. This resulted in a success rate of 77% [70]. Although there were no major bleeding complications, three patients underwent major complications, including right ventricular wall hematoma, arteriovenous fistula at the site of cannula insertion, and coronary perforation with hemodynamic compromise [70]. Therefore, despite an improvement in hemodynamics using TandemHeart before and during high-risk PCI, there is no significant improvement in mortality. Data on the use of TandemHeart on high-risk PCI are also reduced to observational studies (Table 1) [19].

In terms of hemodynamic support in the period preceding heart transplantation, Natucci et al. determined that; although short-stay devices (including TandemHeart, Impella, ECMO, and IABP) are mostly used as a bridge to heart transplantation; they hold some disadvantages, such as full patient anticoagulation in the case of TandemHeart and Impella; therefore, they suggested a long-stay device, the HeartWare, as the best option for the period preceding heart transplantation [40]. Moreover, they found the residual interatrial communication in TandemHeart an additional limitation (Table 1) [40]. As mentioned before, anticoagulant therapy before TandemHeart placement is necessary, and contraindications include ventricular septal defect (as it can cause right-to-left shunt), severe peripheral vascular disease, aortic insufficiency, and intra-atrial thrombus. Concurrent right ventricle failure is also a contraindication and the addition of a right ventricular device may be needed [64].

Finally, with respect to life-threatening arrhythmias such as ventricular tachycardia (VT), TandemHeart is the second most used pVAD after the Impella device in protected VT ablation [71]. Advantages of pVADs’ implantation include the maintenance of end-organ perfusion during VT for longer periods, which require fewer terminations of VT (anti-tachycardia pacing), thus increasing the likelihood that VT ends during ablation [25]. Although these advantages have not been shown to prevent VT recurrence, in-hospital/30-day mortality or procedural success compared to control patients suggested that patients receiving pVADs during protected ablation might experience a reduced risk of long-term mortality [71,72]. Studies also demonstrate that preemptive implantation of Impella or TandemHeart in patients with systolic heart failure can prolong the time on VTs and induce a greater number of VTs [71,72]. Furthermore, a meta-analysis comprising 69 patients, demonstrated that the delayed removal of pVADs in patients undergoing catheter ablation of ventricular tachycardia is associated with increased 90-day mortality [41]. In order to establish conclusive evidence regarding pVADs’ role in systolic heart failure patients undergoing VT ablation, randomized studies are urgently needed on patient selection, device selection, risk stratification, and cost-effectiveness [68].

##### Impella Family Devices

The Impella devices consist of a catheter inserted through femoral access to the left ventricle, crossing the aortic valve [8]. This catheter pumps oxygenated blood from the left ventricle to the ascending aorta via an integrated transvalvular axial pump (Figure 2) [8]. Different models are available depending on the supply they provide: Impella 2.5 (2.5 L/min flow), Impella CP (4 L/min flow), and Impella 5.0 (5 L/min flow) [8]. A recent systematic review conducted by Munoz et al demonstrated the efficacy of the Impella device in improving survival in cardiogenic shock despite the etiology [9]. Moreover, this device has been demonstrated to increase coronary microcirculation while lowering the workload burden in the left ventricle [9]. When comparing the management of the Impella device with IABP in acute myocardial infarction-related cardiogenic shock, the Impella device showed better outcomes, as there was a lower amount of troponins and creatine phosphokinase when using the Impella device [73]. Furthermore, patients previously treated with the Impella device at six months showed a better ejection fraction than those treated with IABP. This demonstrates minor muscle injury and the best recovery [73]. In this respect, Impella PC showed better outcomes for managing cardiogenic shock as a consequence of AMI compared to Impella 2.5, and thus is more beneficial in patients with severe cardiogenic shock [74]. Another study also demonstrated improved renal outcomes in patients with AMI-associated cardiogenic shock, with the Impella device maintaining adequate renal circulation [75]. In general terms, Impella devices are the most widely used, along with IABP and ECMO. Theoretically, they possess the most favorable characteristics for application in the context of high-risk PCI [39,76]. Due to this, the US Food and Drug Administration approved, back in 2015, the use of Impella 2.5 in high-risk PCI, whether it is elective or urgent [21]. However, despite this approval, the most recent systematic reviews and meta-analyses show no improvement in mortality when using the Impella device compared to non-mechanical circulatory support or control patients (using IABP and medical therapy) [13,14,77]. Accordingly, patients treated with the Impella device had increased major bleeding compared to control groups, which was the leading complication in all these studies [14,15,77]. Although the Impella device holds relevant advantages such as superior hemodynamic support than IABP, small-size cannula, and facility for bed position adjustment, etc, the complication of major bleeding due to anticoagulation before the procedure remains and amplifies its limitations (Table 1) [64].

##### Novel Device: Protek Duo

The Protek Duo veno-venous cannula consists of two different lumens with a wire-reinforced cannula body (31-Fr) [42]. Protek Duo is inserted percutaneously through the internal jugular vein. It is specifically placed in the heart so that the inflow lumen is situated in the right atrium, and the outflow lumen in the pulmonary trunk, thus allowing blood to move from the right atrium into the pulmonary artery (Figure 2). This improves oxygenation and relieves both the right atrium and ventricle of the heart. Thus, it also helps prevent complications related to arterial cannulation in patients waiting for lung transplantation with end-stage lung disease, preventing peripheral arterial cannulation or central cannulation [42,46].

The Protek Duo cannula has been shown to offer the advantage of minimally invasive percutaneous full right heart support when combined with the TandemHeart pump, in recent studies (Table 1) [78]. In addition to this, Khanpey et al. also described a minimally invasive temporary biventricular full-flow support system using two ProtekDuo cannulas. It consisted of a modified shortened 29Fr Protek Duo cannula (cut 10.5 cm shorter from the distal end making it a single port) that was passed transapical, draining the left ventricle and ejecting into the ascending aorta on the left side, and a normal 29Fr ProtekDuo cannula inserted percutaneously through the right internal jugular vein on the right side. The cannula was placed in the Seldinger technique over a Swan–Ganz catheter into the pulmonary trunk [79].

The Protek Duo cannula has the benefit of having two lumens; however, it has some drawbacks. One of them is that it drains only from the superior vena cava, making it harder to place it correctly in shorter patients. Additionally, it is more expensive than a standard ECMO cannula; the cost is estimated to be more than USD 20,000 [47].

In a recent systematic review, the Protek Duo cannula was found to be more efficient in increasing survival and reducing complications when used as part of the percutaneous right ventricular assist device (tpRVAD) system, rather than tpRVAD alone [48]. For inclusion, 7 studies with 127 patients in total were eligible. These studies included patients with acute right ventricular failure (aRVF) from a variety of causes. In 2 studies, the patient survival rate to discharge was between 60% and 85.2%, and a 30-day survival rate between 60% and 85.2% was reported in 4 studies. Device-related and non-device-related complications were low [48].

In conclusion, the patients that were treated with a right ventricular assist device (RVAD) using the Protek Duo cannula had comparably better survival rates and fewer complications than other tpRVAD systems [48]. This supports the fact that the Protek Duo cannula is a safe and feasible treatment for patients who may develop acute right heart failure after left ventricular assist device (LVAD) implantation [46].

#### 5.1.2. Devices That Do Not Unload the Heart but Provide Hemodynamic Support

##### VA-ECMO

The use of veno-arterial extracorporeal membrane oxygenation (VA-ECMO) is well documented and widely used, especially in pediatric patients and with cases of respiratory failure [12,80]. A 21–25 Fr cannula is inserted into the right atrium which drains deoxygenated blood. This blood enters into the circuit and undergoes gas exchange in the membrane oxygenator. The blood finally returns via a 15–19 Fr catheter to the systemic circulation via the arterial cannula in the iliofemoral artery (Figure 2) [64]. ECMO technology can be divided into two different categories: venovenous and veno-arterial ECMO. Although both devices provide respiratory support, only VA-ECMO provides circulatory support for CS [64]. Because VA-ECMO simultaneously provides circulatory and respiratory support, it is a suitable treatment option for patients suffering from biventricular failure (Table 1) [19]. The outcome when using VA-ECMO for the management of patients undergoing cardiogenic shock highly depends on the etiology of heart failure, and is usually used in refractory cardiogenic shock cases [81,82]. Cardiogenic shock treated with VA-ECMO and originated by myocarditis or primary graft failure after a heart transplant has excellent results [81,82]. Patients with severe cardiogenic shock originated by AMI with ST-elevation but promptly treated with PCI can also have good outcomes [83]. However, the mortality rate for cardiogenic shock patients after cardiotomy that cannot be weaned off with cardiopulmonary bypass is very high [16]. Although traditionally antithrombotic therapy was necessary prior to VA-ECMO treatment, it is no longer mandatory due to improvement in the biocompatibility of the components, which has diminished mortality and thrombotic events [84]. Concerning VA-ECMO use in life-threatening arrhythmias, a recent systematic review comprising 7 studies and 867 patients concluded a short-term mortality of 15% when using VA-ECMO during ventricular tachycardia (VT) ablation and refractory VT, cardiac arrest, and acute heart failure. The same study also concluded that additional data regarding patient selection, optimization of the surgical procedure, and clinical outcomes are necessary for assessing the efficacy of this strategy [85]. With regards to the use of VA-ECMO in high-risk PCI, a single-center registry comprising 14 patients demonstrated procedural success in 12 patients (85.7%) [44]. The study ultimately concluded that using VA-ECMO in high-risk PCI is a feasible treatment option, although more extensive studies needed to be conducted [44]. Lastly, a meta-analysis that comprised 41 patients undergoing high-risk PCI, demonstrated no difference in the outcomes when using prophylactic Impella versus VA-ECMO in patients undergoing high-risk PCI [32]. Therefore, the use of VA-ECMO in high-risk PCI has been demonstrated to have good results, although more research is necessary to confront it with other cardioprotective devices [18].

##### IABP

The Intra-Aortic Balloon Pump (IAPB) is a simple, cost-effective method used to assist the heart indirectly by decreasing the afterload and increasing the diastolic aortic pressure. This results in better perfusion of peripheral organs and coronary blood flow, increasing cardiac output by up to one liter per minute [50,51]. The balloon inflates during diastole synchronously with aortic valve closure, and rapidly deflates before the onset of the systole (Figure 2) [50]. Zhang et al. (2022) found, in a meta-analysis reviewing 4416 articles, that IABP is more effective for the treatment of cardiogenic shock, reducing the incidence of 30-day mortality compared with VA-ECMO and Impella [17]. The study by Alushi et al. (2019) states that IABP has been downgraded in guidelines after the IABP-SHOCK II trial failed to prove any mortality benefit over medical therapy alone [86,87]. Common risks when using MCS include bleeding and strokes. In both cases, IABP was found to perform better than Impella and TandemHeart, because of the non-mandatory use of unfractionated heparin for IABP. However, compared to other MCS devices, the risk of bleeding is significantly higher [87]. Nevertheless, the same study shows the SUCRA values of the different MCS devices on a scale from 0 to 1, where IABP achieved the worst score of the 6 that were compared (IABP, Impella, TandemHeart, ECMO, ECMO + IABP, and ECMO + Impella) [87]. Guidelines assign a Class IIa recommendation to IABP for stage D congestive heart failure patients [51,86]. Naqvi et al. (2018) also describe IABP use for congestive heart failure as a “bridge to recovery or bridge to decision”; mentioning the risks involved in IAPBs for patients undergoing heart transplant; including the significantly higher serum creatinine, lower BMI, and more functional impairment requiring full assistance with activities of daily living, compared to LVAD [51]. Other common complications may include major acute limb ischemia, balloon leak, IAPB failure, and death [50,51].

Nowadays, IABP is not recommended in most procedures of interventional cardiology, due to its poor performance in patients with poor left ventricular function undergoing coronary artery bypass surgery and cardiogenic shock (Table 1). Moreover, numerous trials in interventional cardiology regarding trans-myocardial laser, thrombus extraction devices, and bivalirudin have failed to show benefits. Mishra (2018) implies there could still be some benefit if IABP was employed just prior to actual ventricular decompensation for prophylactic purposes [52].

##### Surgical BiVAD

The Bi-Ventricular Assist Device (BiVAD) is a form of mechanical support used in patients with chronic or acute biventricular failure as a bridge to a transplant or recovery [11,53]. The installation of this device requires a surgical procedure, starting with a sternotomy and division of the pericardium, then initiating the cardiopulmonary bypass to start cannulation of the BiVAD. The optimum site of cannulation is away from the interventricular septum and coronary vessels, with the guide of a transesophageal echocardiography (Figure 2) [11]. Continuous flow devices are preferred over pulsatile flow devices for BiVAD due to the smaller size that reduces surgical trauma, the higher durability, and energy efficiency [54]. Chen et al. (2022) conclude after a 5-year-long study that BiVAD could be a salvage treatment for patients with severe cardiogenic shock if combined with extracorporeal life support, by correcting organ low-perfusion and allowing sufficient time to bridge patients to recovery or heart transplantation [88]. A common complication in ventricular assist devices is ventricular arrhythmia, associated with a higher mortality (Table 1) [88]. Lin et al. (2019) quantified the complications, concluding that 46% of their population experienced ventricular arrhythmias after the BiVAD placement, and analyzed the probable causes, including inotrope use (56%), suction events (12%), hypokalemia (7%), and right ventricle assist device thrombosis (5%); 20% were not associated with any triggers. Nevertheless, the most common adverse events overall were major bleeding and hospital readmission [89]. Ruhparwar et al. (2019) describe a different approach to BiVAD, with a model promising rapid extubation thanks to the less-invasive procedure, avoiding the need for a sternotomy [90]. This concept consists of a full-flow MCS system for cardiogenic shock patients due to biventricular heart failure, named ECPELLA 2.0, using an Impella device as an LVAD combined with a TandemHeart/ProtektDuo system as an RVAD. Early mobilization facilitates a faster recovery while reducing the chances of limb ischemia or groin vessel injuries [90].

### 5.2. Myocardial Cooling Devices and Techniques

#### 5.2.1. The Topical Myocardial Cooling Device

Many cardiac-preserving techniques have been used since the inception of cardiac surgery. However, cardioplegic arrest and hypothermia are the mainstay approaches to protecting the heart during cardiac surgery [91]. Hypothermia provides cardioprotection, mainly by slowing down the metabolism of cardiomyocytes. This reduces their demand for oxygen and energy consumption [91,92]. A Topical Cooling Device (TCD) is an example of devices that are used to achieve hypothermia during cardiac surgeries. It is made up of a closed system that utilizes sterile saline solution at the temperature of 4 °C within the pericardium to maintain cardiac hypothermia during cardiac surgery. TCD is a silastic, double-membrane blanket that is designed in such a way that it can be easily fitted outside of the heart during cardiac surgery. This blanket is then connected to the closed hypothermic sterile normal saline solution that provides a continuous flow of fluid ranging from 200 to 350 mL/min [4].

This is the latest simpler method of deploying hypothermia that can possibly be used in addition to cold cardioplegia to achieve the following goals: (1) rapid termination of heartbeat after cross clamping of aorta and (2) rapid achievement of hypothermia with temperatures ranging between 10 °C and 20 °C [4].

In an experimental study conducted by Garcia-Rinaldi et al. (1981) on patients who underwent aortocoronary bypass, various patterns of myocardial cooling and rewarming were studied after administering 1 liter of cold (4 °C) cardioplegic sterile saline solution [93]. These patterns were comparatively analyzed, with the myocardial cooling and rewarming patterns associated with continuous topical hypothermia. Rapid myocardial hypothermia was achieved right after the administration of 1 liter of cardioplegic solution. However, myocardial rewarming can be altered to sustain myocardial hypothermia (<20 °C) by manipulating systemic temperature, use of TCD, and method of cannulation [93].

Regarding TCD, it was found that it functions best when the heart system is completely cut off and isolated from the systemic circulation, i.e., using occlusive vena caval cannulae and left ventricular sump. TCD, however, did not maintain cardiac hypothermia when the patient was maintained under systemic normothermia, in contrast to the moderate systemic hypothermia (≤30 °C) in which TCD effectively maintained cardiac hypothermia after deployment of the double caval cannulation technique. Thus, TCD works best when the systemic temperature is decreased [93].

The Topical Cooling Device has also been constructed for the pediatric population, with five different sizes for accommodating any size of the pediatric heart. In research conducted by Villamater et al. (1986), a pediatric TCD was routinely used for three consecutive years without any frequent complications of diaphragmatic paralysis. They stated that this pediatric TCD is efficacious and easy to use in all pediatric cardiac surgeries [10].

In another comparative study, by Nikas et al. (1998), 505 hospitalized patients undergoing coronary artery bypass graft were selected at the University of Alabama, to analyze the effectiveness of topical cardiac hypothermia and its association with pulmonary complications. To their surprise, topical hypothermia did not provide any cardioprotective benefit in coronary bypass patients. However, a significant increase in diaphragmatic paralysis and pleural effusions was found. This indicated that topical cardiac hypothermia is not an effective method for coronary artery bypass patients [94]. However, newer studies recommend the use of myocardial cooling devices in patients undergoing cardiopulmonary bypass, as it can significantly improve outcomes following cardiac arrest (Table 1) [95].

In an experimental study by Rosenfeldt et al. (1982)*,* the hypothermic cardioplegic arrest was ineffective, inadequately cooling the anterior left ventricular wall or interventricular septum in the experimental dogs. However, the spray system for cardiac hypothermia proved to be highly efficacious in producing myocardial hypothermia ranging from 6 °C to 12 °C. Thus, it was concluded that the spray system is superior to the topical cooling device system in achieving cardiac hypothermia [96].

#### 5.2.2. Topical Neck Cooling

Acute Myocardial Infarction, whether NSTEMI or STEMI, is a highly serious and emergent condition that can prove fatal if prompt cardiac revascularization is not achieved in a timely manner. This is because early revascularization is the best and only possible approach to reduce post-myocardial infarction complications such as scarring, aneurysm, reperfusion injury, etc. [97]. During recent advancements in the medical and health industry, many methods have been invented to slow down myocardial injury before revascularization is achieved in myocardial infarction patients. Promising results have been shown by the use of systemic hypothermia to slow pre-revascularization myocardial injury; however, this technique demands high resources and is not conducive in emergency hospital settings. For this reason, Topical Neck Cooling (TNC) therapy (which is also highly effective) has been devised to be implemented while the patient is en route to the catheterization lab for revascularization [97].

In the latest research published by Zhang et al. (2022), the infarct-attenuating effect of TNC was found to be comparable to systemic hypothermia. Profound attenuation of infarct size was seen with TNC during myocardial ischemia, thus exerting a cardioprotective effect without the need for a change in core body temperature [98].

It has also been proven in animal models that TNC can provide a beneficial effect in a way that it can prolong the survival of rats suffering from severe intra-abdominal sepsis. This effect is due to the inhibition of systemic proinflammatory states by stimulating anti-inflammatory vagal nerve pathways [99]. This can possibly be useful in that postsurgical infections and/or sepsis can be prevented by the utilization of such a technique. However, human trials are yet to be conducted [99].

### 5.3. Transcutaneous Vagus Stimulation

The vagus nerve is an important cranial nerve that plays crucial physiological and homeostatic roles in regulating cardiac function. There is a well-established link between heart rate variability and vagal tone. It is the vagus nerve that maintains the heart rate in its normal range by preventing tachyarrhythmias [100]. Because of the rich innervation of the heart with the vagus nerve, vagus nerve stimulation has been proposed to be a potential therapy for curing cardiovascular disorders such as cardiac arrest, cardiac arrhythmia, acute myocardial infarction, and probably a stroke [57].

Transcutaneous vagus nerve stimulation (tVNS) is a non-invasive FDA-approved therapy that is mainly used for the treatment of either depression or epilepsy (Table 1). It involves the application of electrical currents at the selected desired locations via the surface electrodes, so that the vagus nerve can be stimulated. The most common area involves the auricular branch of the vagus nerve and the cervical branch of the vagus nerve in the neck region where the electrical currents are deployed [101].

A recent preclinical study by Sun et al. in rats found that the success rate of return of spontaneous circulation (ROSC) after an induced cardiac arrest is far greater (90.91%) with vagus nerve stimulation plus CPR, compared to just CPR alone (83.33%) [22]. It was also found that fewer defibrillator shocks were required for ROSC when the vagus nerve was stimulated. The possible mechanism could be the dampening of sympathetic flow to the heart during resuscitation attempts via defibrillation. Another group of researchers in 2014 found an interesting result when vagus nerve stimulation directly modulated the function of the left ventricle in pigs and sheep in such a way that it caused an increase in ventricular action potential duration and effective refractory period, thus relaying a possible therapeutic role in post-myocardial infarction ventricular arrhythmia treatment [56].

Another highly useful role of tVNS has been found in inducing asystole for possible novel use in cardiac surgeries for cardioplegia. Research shows that tVNS can induce intermittent cardiac asystole that can then be used as an “on-off” switch for performing cardiac surgeries or minimally invasive procedures [56].

### 5.4. Pressure-Controlled Intermittent Coronary Sinus Occlusion

Pressure-controlled intermittent coronary sinus occlusion (PiCSO) is a mechanical-catheter-based device that increases the mean coronary sinus pressure and coronary sinus pulse pressure intermittently post PCI. This protects the microvasculature by increasing its perfusion, salvaging more myocardium, and reducing complications such as heart failure or death [58,59]. PiCSO is limited to treating anterior STEMI [59]. The system consists of an 8F balloon-tipped catheter placed in the coronary sinus, controlled by a console; an algorithm constantly measures the coronary sinus pressures and coordinates the balloon inflation and deflation cycles [58]. Egred et al. (2020) obtained the important conclusion that patients who received PiCSO after reperfusion have a significantly smaller infarct size at 5 days and no procedural or device-related adverse effects [59]. A year later, Scarsini et al. (2021) reached four key results in the PiCSO study:

1. PiCSO immediately improved microvascular function after PCI in STEMI patients;
2. PiCSO positively influenced coronary microcirculatory vasodilation;
3. PiCSO-assisted PCI demonstrated a smaller infarct size at 6 months;
4. PiCSO showed promising results in treating inferior STEMI [102].

The effects of PiCSO in the heart reach a molecular level, by increasing relevant microRNA and transcription factors favoring cardiac regeneration by acting on cardiomyocytes and cardiac fibroblasts [60]. Further investigations are being conducted on PiCSO, yet there is still a lot to learn from this therapy; therefore, further investigation on this topic will be very beneficial (Table 1) [58,59,60,102].

### 5.5. Supersaturated Oxygen Therapy

Super Saturated Oxygen (SSO_2_) therapy is a proprietary medical technology that creates a highly oxygenated saline solution and combines it with the patient’s arterial blood to provide focal hypoxemic oxygen therapy to ischemic tissues, and restore microvascular flow. SSO_2_ therapy is normally used for the treatment of acute myocardial infarction; however, it also complements PCI and is designed to minimize myocardial damage. SSO_2_ has been shown in an FDA-sanctioned trial to reduce infarct size of the left ventricle from approximately 25–27% to about 19%, which can be attributed to an attenuation of reperfused myocardial infarction with improvement in microvascular blood flow [103].

One of the major advantages of SSO_2_ over other methods for reducing infarct size during acute STEMI treatment in the cath-lab is that it can be started after successful revascularization, without delaying primary PCI. This is in contrast to other methods such as LV unloading by Impella or therapeutic hypothermia, which may require additional steps or procedures that could delay the primary treatment (Table 1) [61].

The IC-HOT study published in 2020 evaluated the clinical outcome of intracoronary SSO_2_ therapy after primary PCI in patients with anterior STEMI. There were 100 patients treated with SSO_2_ who had successfully undergone PCI of an occluded left anterior descending coronary artery without cardiogenic shock [104]. These results were compared with a propensity-matched control group of similar patients with anterior STEMI, enrolled in the INFUSE-AMI trial. Baseline and postprocedural characteristics were similar in the two groups except for pre-PCI thrombolysis in myocardial infarction 3 flow, which was less prevalent in patients treated with SSO_2_ (9.6% vs. 22.9%, *p* = 0.02) [104]. All-cause mortality, driven by cardiovascular mortality and a beginning heart failure (HF) or HF hospitalization was each individually lower in SSO_2_-treated patients in comparison with the ones that were not [104].

Lastly, a study regarding updates on cardioprotective strategies for STEMI was a multicenter study of 29 patients with acute STEMI reperfused with primary PCI, in which hypoxemic blood was infused for 60 to 90 min, right after proximal patency was established. The hypoxemic reperfusion was accomplished successfully in all of the patients, without therapy-related adverse events [62,105]. An echocardiographic study carried out on the patients showed an improvement in the wall motion score index and a trend toward an increase in mean left ventricular ejection fraction (LVEF) at 24 hours; approximately 48.6% improved immediately after angioplasty, against a 51.8% improvement after 24 hours. After a month, the wall motion score and the LVEF improved by about 54.4%, and at 3 months it improved by 56% [62]. These clinical studies have been able to demonstrate that the SSO_2_ therapy is a safe and successful agent to apply with reperfusion, in order to reduce infarct size, preserve cardiac function, reduce adverse LV remodeling, and, more likely, to improve most clinical outcomes in patients with acute anterior STEMI reperfused within 6 hours of symptoms as possible [62].

In addition to what has been mentioned before, a major advantage of this therapy compared with other treatments is that the reperfusion is not being delayed; future studies will have to be focused on the outcomes of SSO_2_ therapy in STEMI and further explore its different effects [62].

## 6. Newer Therapeutic Techniques in High-Risk Populations (Cardiogenic Shock and PCI)

Along with the new techniques under investigation, it has been suggested that the combination of existing or novel techniques can reduce patient mortality when treating profound CS, or give further cardioprotection for percutaneous surgery in high-risk populations. A recent meta-analysis counting 2573 patients and 9 manuscripts found that when CS patients received ECMO combined with IABP, their in-hospital survival rate was significantly higher than when they received ECMO alone, and, thus, concluded that the combination of ECMO and IABP could significantly improve the survival rate compared to using ECMO alone (Table 1) [20]. Moreover, the combination of Impella and VA-ECMO has been associated with increased survival in patients with CS, despite increased hemolysis rates and the need for renal replacement therapy. Furthermore, major bleeding and cerebrovascular events were not increased (Table 1) [21].

Other forms of combinations, such as the combination of pharmacological therapy (catecholamines, and particularly low doses of norepinephrine) with Impella CP in severe CS, have also been shown to improve oxygen delivery and cardiac work. Nonetheless, it is advised to have great caution when using phenylephrine during treatment CS [22].

With regards to improving cardioprotection during PCI, Udesen et al. (2020) demonstrated in an animal model that a combination of mild hypothermia (MH) (defined as a temperature of 32 °C to 35.9 °C) with selective coronary venous autoretroperfusion (delivering oxygenated blood through the coronary venous system to the ischemic myocardium) could preserve cardiac function and reduce myocardial infarct size [22]. This could constitute a better approach compared to currently available devices (VA-ECMO, Impella, and TandemHeart), as they offer little cardioprotection when it comes to obstructed coronary arteries compared to MH and autoretroperfusion [22]. Although this approach looks promising, more research, particularly first-human translation, is needed [106].

## 7. Conclusions

Cardioprotective devices have shown promising results in the setting of acute heart failure and cardiogenic shock; however, their use in the surgical setting has only recently started to become clinically relevant. The principle of LV unloading has shown promising results in preserving myocardial function and integrity at different levels, especially in high-risk patients. Similarly, the use of pVADs has been shown to significantly improve cardiac output and mean arterial pressure; however, no associated reduction in patient mortality was achieved. The combination of pVADS (Impella + VA-ECMO or IABP+ ECMO) has shown promising results in mortality reduction, but more research is needed. Other approaches such as cardioplegia and vagal stimulation have also shown positive outcomes; however, the literature supporting their use in high-risk patients undergoing cardiac surgery or PCI remains limited. To significantly reduce mortality related to cardiac surgeries and procedures, new techniques, targets, and approaches are necessary. Human translation will be a crucial step in the advancement of these devices. Finally, more studies highlighting the cost-effectiveness of these devices are needed to guide surgeons in decision making.

## Figures and Tables

**Table 1:** Comparison of Cardioprotective Devices Used in PCI and Cardiac Surgery.

		Uses in PCI and Cardiac Surgery		
		Ventricular Support	Advantages	Disadvantages/Limitations
Devices that provide cardioprotection by improving hemodynamics or providing circulatory support	TandemHeart [39,40,41,42]	Left ventricular support	Hemodynamics improvement before and during PCI	No significant improvement in mortality Data limited to observational studies Need of anticoagulant therapy before placement Invasive device: need of interatrial communication
	Impella family devices [14,42,43]	Left ventricular support Impella RP: right ventricular support	Hemodynamics improvement before and during PCI Small size cannula Approved by the US Food and Drug Administration for high-risk PCI	No significant improvement in mortality Significant major bleeding complications Need of anticoagulant therapy before placement May induce right heart failure
	VA-ECMO [19,32,44,45]	Biventricular support	Provides circulatory and respiratory support, ideal for patients undergoing biventricular failure Some studies show procedural success and no difference in outcomes compared to Impella family devices when used in high-risk PCI	More research is needed to conclude its efficacy in high-risk PCI
	Protek Duo [45,46,47,48,49]	Right ventricular support	Safe and feasible treatment in patients with acute right heart failure resulting from implementing a left ventricular assist device. In conjunction with TandemHeart, may offer up to a month of circulatory support. Minimal invasive percutaneous full right heart support ProtekDuo as a bridge to lung transplant and heart-lung transplant	Efficacy and safety data on this device are limited. Drains only from the superior vena cava, making it harder to place it correctly in shorter patients. More expensive than a standard ECMO cannula (> USD 20,000)
	IABP [50,51,52]	Left ventricular support	Cost-effective method No need for anticoagulant therapy before placement	Poor performance in patients with poor left ventricular function undergoing artery bypass surgery and cardiogenic shock
	BiVAD [11,53,54]	Biventricular support	Good outcomes when used in patients with chronic or acute biventricular failure as a bridge to transplant or recovery Beneficial in patients undergoing right-sided heart failure	Need of sternotomy Ventricular arrhythmias after device placement More research needed to assess its efficacy in high-risk PCI
	IABP+ ECMO [20]	Biventricular support	May reduce mortality when treating profound cardiogenic shock (CS) Hemodynamics improvement before and during PCI	Only small observational studies available, not enough for concluding efficacy. Poor data concerning IABP+ECMO in PCI
	Impella + VA-ECMO [21]	Biventricular support	May reduce mortality when treating profound CS Hemodynamics improvement before and during PCI	Only small observational studies are available, which is not enough to conclude efficacy. Poor data concerning Impella+ECMO in PCI
Devices that provide cardioprotection by the preservation of myocardial properties	Myocardial cooling devices [4,10,55]	NA	Used in people after induced cardiac arrest following surgery. May minimize ischemia–reperfusion injury, thereby improving cardiac surgery outcomes after cardiac arrest. Efficacious and easy to use in all pediatric cardiac surgeries. Key therapy in patients undergoing cardiopulmonary bypass surgery requiring cardiac arrest	Risk of widespread intravascular crumpling Although it has been shown to have good results in clinical trials, more research is needed to show the same results in human trials
Other approaches	Transcutaneous vagus stimulation [56,57]	NA	Non-invasive therapy Can induce intermittent cardiac asystole and can be used as an “on-off” switch for performing cardiac surgeries	More research is needed to assess all the advantages and risks for its use in cardiac surgery [57]
	Pressure controlled intermittent coronary sinus occlusion [58,59,60]	NA	Increases the mean coronary sinus pressure and coronary sinus pulse pressure after a PCI PiCSO-assisted PCI has demonstrated smaller infarct size after 6 months	Limited to treating anterior ST-elevated myocardial infarction More research needed
	Supersaturated oxygen therapy [61,62,63]	NA	Reduces infarct size. Improves reperfusion injury. Reduces endothelial edema and capillary vasodilation. Can be started 5 min after successful revascularization, without delaying primary PC	Relatively new therapy with unknown long-term outcomes

## Data Availability

No new data generated.
